# Warning factors of metachronous uterine cancer in patients with breast cancer: a real-world nationwide cohort study

**DOI:** 10.1016/j.gore.2025.101732

**Published:** 2025-04-05

**Authors:** Yi-Jou Tai, Chung-Chen Lee, Yong-Chen Chen, San-Lin You, Ying-Cheng Chiang

**Affiliations:** a Department of Obstetrics and Gynecology, National Taiwan University Hospital, Taipei, Taiwan; b Department of Obstetrics and Gynecology, College of Medicine, National Taiwan University, Taipei, Taiwan; c Data Science Center, College of Medicine, Fu-Jen Catholic University, New Taipei City, Taiwan; d School of Medicine, College of Medicine, Fu-Jen Catholic University, New Taipei City, Taiwan; e Department of Obstetrics and Gynecology, National Taiwan University Hospital, Hsin-Chu Branch, Hsin-Chu City, Taiwan

**Keywords:** Breast cancer, Tamoxifen, Metachronous uterine cancer, Warning factors

## Abstract

**Objective:**

This study aimed to explore the risk factors for metachronous uterine cancer, including age at diagnosis, stage, body mass index (BMI), medical history and tamoxifen treatment in women with breast cancer to enable tailored risk-based gynecological surveillance.

**Methods:**

A nationwide population-based cohort study was conducted. Data were retrieved from the Taiwan National Health Insurance Research Database and Taiwan Cancer Registry from 2011 to 2019. The risk of metachronous uterine cancer was analyzed by Cox regression model.

**Results:**

There were 307 patients identified with uterine cancer among 114,906 patients with breast cancer. The incidence was 37.46 cases per 100,000 person-years among tamoxifen non-users (69 cases per 184,207 person-years). Among tamoxifen users (n = 58,227), the incidence rates were 105.52 cases (79 cases per 74,869 person-years) for <1 year of use, 111.62 cases (111 cases per 99,444 person-years) for 1–3 years, 58.19 cases (31 cases per 53,276 person-years) for 3–5 years, and 62.12 cases (17 cases per 27,366 person-years) for ≥5 years. The hazard ratio (HR) for uterine cancer was 3.06 (95 % confidence interval [CI] 2.14–4.39), 3.03 (95 % CI 2.15–4.28), 1.61 (95 % CI 1.01–2.57), and 1.77 (95 % CI 1.00–3.13) for patients on tamoxifen for <1, 1–3, 3–5, and ≥5 years. High BMI (≥25 kg/m^2^) was associated with an increased risk (HR 2.46, 95 % CI 1.07–5.64). Abnormal uterine bleeding was a significant predictor regardless of the clinical or sonographic diagnosis of endometrial lesions.

**Conclusion:**

Routine ultrasound is not recommended for the detection of metachronous uterine cancer, and gynecologic investigations should focus on breast cancer patients with high BMI, tamoxifen use, and abnormal uterine bleeding.

## Highlights

• The increasing incidence of metachronous uterine cancer is an important issue for women with primary breast cancer.
• High BMI, tamoxifen use and abnormal uterine bleeding were warning factors of metachronous uterine cancer.
• Gynecologic investigations for metachronous uterine cancer should focus on patients with warning factors.

## Graphical abstract

**Figure:**
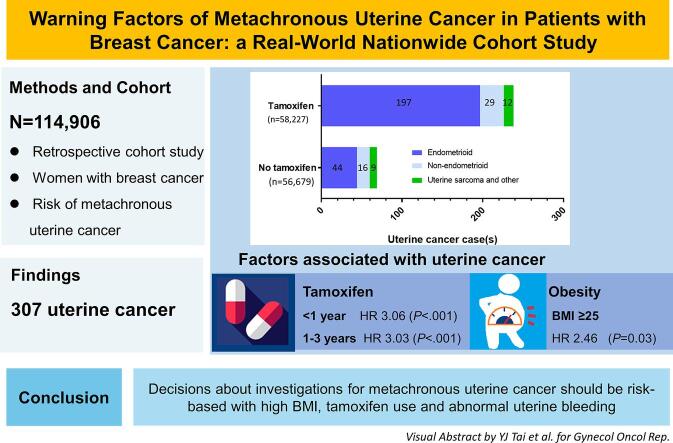

## 1 Introduction

Breast cancer is the most commonly diagnosed cancer in women, with the highest incidence rates observed in France, Australia, North America, and Northern Europe. However, rapid increases in breast cancer incidence have been observed in Asian countries. (Sun et al., 2023, Bray et al., 2024) In Taiwan, secular trends in the incidences of breast and uterine cancers have been increasing with a strong birth effect, and the largest increase was observed for ductal and lobular carcinomas of the breast and endometrioid carcinomas of the uterus, which indicates that estrogen-related cancer plays an essential role in young women. (Tai et al., 2023, Lin et al., 2012) The projected age-specific incidence of breast cancer for women aged 50–59, 60–64, and older than 65 years exhibits a plateau in 2031, 2033, and 2035, respectively. For women younger than 50 years, the incidence is estimated to plateau around 2017 to 2027. (Chen et al., 2022) Tamoxifen is commonly used as an adjuvant endocrine therapy for estrogen receptor-positive breast cancer in premenopausal and selected postmenopausal patients. The increasing incidence and younger peak age of breast cancer occurrence suggest that the proportion of patients receiving tamoxifen has gradually increased. In clinical practice, it is important to select patients at greater risk of developing metachronous uterine cancer.

Compared with the general population, patients with breast cancer have an increased risk of developing subsequent cancers. Cancers of the endometrium, ovary, thyroid, lung, stomach, and colon have been reported more frequently as second primary cancers. (Sung et al., 2021, Molina-Montes et al., 2015) The elevated risk of second primary cancers is likely attributed to genetics, shared risk factors such as lifestyle factors, environmental exposures, or the late effects of treatment. (Molina-Montes et al., 2015, Molina-Montes et al., 2013, Brantley et al., 2024) The higher risk of uterine cancer after hormone receptor-positive breast cancer may partly reflect the use of tamoxifen therapy. Tamoxifen use reduces the risk for subsequent breast cancer, and the ATLAS study had shown that adjuvant tamoxifen for 10 years versus 5 years leads to a greater reduction in breast cancer recurrence. (Lazzeroni et al., 2023, Davies et al., 2013) However, Ryu *et al*. reported a higher incidence of endometrial cancer in tamoxifen users (2.01 per 1000 person-years) than in non-users (0.45 per 1000 person-years), and tamoxifen use was associated with increased risks of endometrial polyps, hyperplasia, and cancer. (Ryu et al., 2022) The risk of uterine cancer persisted after cessation of tamoxifen use. (Chu et al., 2019) Therefore our study aimed to identify warning factors for metachronous uterine cancer in women with breast cancer, aiding clinicians in selecting appropriate patients for invasive diagnostic procedures, including endometrial evaluation (aspiration, biopsy, and polypectomy) and dilation and curettage (D&C).

## 2 Methods

### 2.1 Data Source and study population

This study was reviewed and approved by the Institutional Review Board of Fu Jen Catholic University (IRB no: C110216). Data were retrieved from the Taiwan National Health Insurance Research Database (NHIRD) and Taiwan Cancer Registry. The Taiwan National Health Insurance program covers more than 99 % of the 23.7 million residents of Taiwan, and NHIRD contains health care information, including International Classification of Diseases (ICD) codes, drug prescriptions, medical procedures, outpatient visits, and hospitalizations. In this study, newly diagnosed cases of breast and uterine cancers were identified using the Taiwan Cancer Registry, and treatment information was collected from the NHIRD. Death certification profiles were used to censor loss to follow-up and death. The diagnosis was based on the ICD; ICD-9-CM codes were used for 2015 and earlier, and ICD-10-CM codes were used for 2016 and later. The diagnosis of primary cancer was based on the ICD-O-3 and classified using histological codes (ICD-O-3 Morphology, M-codes) (Supplementary Table 1).

Data of patients with breast cancer between January 1979 and December 2019 were extracted from the Taiwan Cancer Registry Annual Report. Since some cases before 2010 lacked data on body weight (body mass index [BMI]) and histological details, such as hormone receptor status (ER, PR, and HER2), breast cancer cases diagnosed before 2010 were excluded from the study. The exclusion criteria were as follows: patients diagnosed with breast cancer before 2010, those with any history of cancer (excluding carcinoma in situ [CIS]) prior to the breast cancer diagnosis, and those with a history of hysterectomy. Only patients diagnosed from 2011 onward were included in the following steps of analysis. Patients were also excluded from the analysis if the interval between the diagnosis of breast cancer and uterine cancer was less than 180 days, if they had records of tamoxifen use before the diagnosis of breast cancer, or if they had an incorrect death certificate. The histologic subtypes of uterine cancers included endometrioid adenocarcinoma, non-endometrioid adenocarcinoma, uterine sarcoma, and other carcinomas, defined by histological codes other than those mentioned above. (Tavassoeli and Devilee, 2003) Eligible patients with breast cancer were followed up until the time of invasive uterine cancer diagnosis, date of death, or until December 31, 2019, whichever came first. Patients were followed from the date of breast cancer diagnosis until December 31, 2019.

Uterine cancer incidence and dates of diagnosis were determined using ICD-9 code 182 and ICD-10 code C54 after data linkage with the National Cancer Registry and death profile. Uterine cancer diagnoses were obtained from the national pathology-based cancer registry. The follow-up duration for each patient was calculated as the time from the date of breast cancer diagnosis to the date of uterine cancer diagnosis, date of death, or the end of the study period (i.e., December 31, 2019), whichever occurred first. The proportion of morphological verification was 93.0 % for all sites. (Chiang et al., 2019) According to the Cancer Registry Annual Report 2021, 99.8 % of breast cancers and 99.6 % of uterine cancer cancers were confirmed by cytology or histopathology. (Administration and Report, 2021).

Breast cancer diagnostic information included the year of diagnosis, age at diagnosis, disease stage, BMI, and treatment methods. All information was obtained after linking to the cancer registration databases, which contained information on when breast cancer was first diagnosed. Medical history (hypertension, diabetes, dyslipidemia, and polycystic ovary syndrome) and gynecological presentation (abnormal bleeding, pain, and endometrial lesions) were based on the ICD codes used to judge the case before entering the study. Gynecologic presentations (abnormal bleeding, pain, and diagnosis of endometrial lesions) were not limited to presentations before or after the breast cancer diagnosis. Endometrial lesions were identified based on ultrasound findings, hysteroscopy, or invasive procedures such as endometrial biopsy. In this study, we included these patient groups in the analysis based on the International Classification of Diseases, Ninth and Tenth Revision, Clinical Modification (ICD-9-CM: 621.0, 621.3, 621.8, 219.1, and 239.5; ICD-10-CM: N84.0, N85.00, N85.01, and N85.8).

In descriptive statistics, continuous variables were expressed as medians and interquartile ranges, while categorical variables were expressed as numbers and percentages. For inferential statistics, the Wilcoxon rank-sum test was used for continuous variables, and the chi-square test and Fisher's exact test were used for categorical variables. When comparing the risk of uterine cancer according to each risk factor, the cause-specific hazard function was used to calculate the hazard ratio (HR). Kaplan–Meier survival curves were plotted, and the log-rank test was used to compare the survival curves between groups. The SAS statistical package (version 9.4, SAS Institute Inc., Cary, NC) was used for analysis. Statistical significance was set at *P* < 0.05.

## 3 Results

### 3.1 Characteristics of study population

**Fig. 1:**
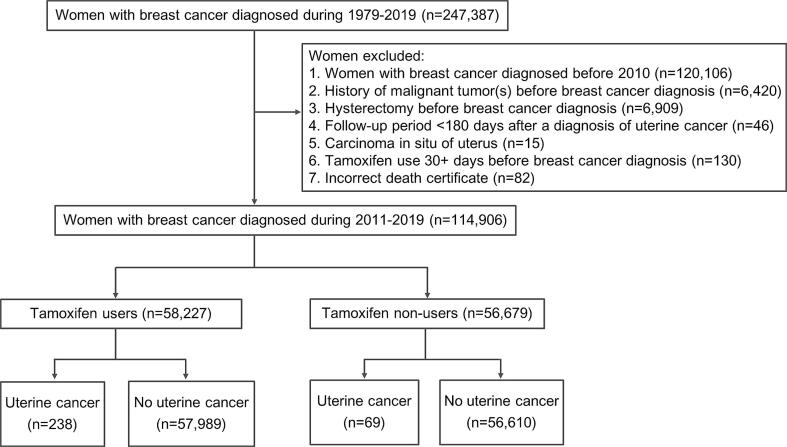
Flow chart of the study population, It illustrated the inclusion and exclusion criteria of participants in the study.

The records of 247,387 patients with breast cancer from January 1, 1979, to December 31, 2019, were retrieved from the Taiwan Cancer Registry (Fig. 1). A total of 114,906 patients with breast cancer were included in the analysis. Within this cohort, we identified 58,227 patients who received tamoxifen (tamoxifen user group) and 56,679 patients who did not (non-user group). The demographic and clinical characteristics of the study population are shown in Table 1 and Table 2**.** The median follow-up duration was 4.26 and 2.77 years in tamoxifen users and non-users.

**Table 1:** Baseline characteristics of 114,906 patients with breast cancer from 2011 to 2019 in Taiwan.

Variables	Patients (n, %)		*P* value
	Tamoxifen users (*n* = 58,227)	Tamoxifen non-users (*n* = 56,679)	
**Year of diagnosis of breast cancer**			0<.001
2011–2013	20,725 (35.6)	12,386 (21.9)	
2014–2016	19,459 (33.4)	18,612 (32.8)	
2017–2019	18,043 (31.0)	25,681 (45.3)	
**Age at diagnosis of breast cancer** (median, 25th-75th percentile)	49 (44–58)	58 (51–65)	0<.001
<40	7,523 (12.9)	3,213 (5.7)	
40–49	23,385 (40.2)	8,555 (15.1)	
50–59	14,743 (25.3)	19,285 (34.0)	
60–69	8,186 (14.1)	16,571 (29.2)	
≥70	4,390 (7.5)	9,055(16.0)	
**Stage of breast cancer**			0<.001
0	10,984 (18.9)	5,349 (9.4)	
I	19,343 (33.2)	14,528 (25.6)	
II	17,414 (29.9)	18,914 (33.4)	
III	5,884 (10.1)	8,869 (15.7)	
IV	2,263 (3.9)	4,803 (8.5)	
Unknown	2,339 (4.0)	4,216 (7.4)	
Treatment of breast cancer*			
None	16 (0.03)	54 (0.10)	0<.001
Surgery	53,707 (92.2)	48,362 (85.3)	0<.001
Radiotherapy	33,070 (56.8)	27,489 (48.5)	0<.001
Chemotherapy	25,866 (44.4)	31,827 (56.1)	0<.001
Hormonal therapy^†^	52,008 (89.3)	25,479 (45.0)	0<.001
Oophorectomy	63 (0.1)	35 (0.06)	0.007
Immunotherapy	38 (0.07)	177 (0.3)	0<.001
Targeted therapy^‡^	6,059 (10.4)	11,249 (19.9)	0<.001
Palliative treatment	1,097 (1.9)	2,477 (4.4)	0<.001
Other treatment	5 (0.01)	9 (0.02)	0.26
Unknown	2,107 (3.6)	3,761 (6.6)	0<.001

Note: *Percentages add to more than 100 % because of overlapping categories. ^†^Hormonal therapy included antiestrogens, LH-RH agonists, and aromatase inhibitors. ^‡^Targeted therapy included HER2-targeted therapy, CDK4/6 Inhibitors, PI3K/AKT/mTOR Inhibitors and PARP Inhibitors.

**Table 2:** Clinical characteristics of 114,906 patients with breast cancer from 2011 to 2019 in Taiwan.

	Patients (n, %)		*P* value a
	Tamoxifen users (n = 58,227)	Tamoxifen non-users (n = 56,679)	
**Follow-up time** (years)	4.26 (2.22–6.54)	2.77 (1.17–5.02)	0<.001
**BMI** (kg/m^2^)	23.3 (21.0–26.2)	23.9 (21.5–26.9)	0<.001
**BMI** (kg/m^2^) b			0<.001
<18.5	2,576 (4.4)	2,111 (3.7)	
18.5–22.9	21,455 (36.9)	17,516 (30.9)	
23–24.9	9,602 (16.5)	9,800 (17.3)	
≥25	17,808 (30.6)	19,903 (35.1)	
Unknown	6,786 (11.6)	7,349 (13.0)	
**History of disease at breast cancer diagnosis**			
Hypertension	15,216 (26.1)	22,776 (40.2)	0<.001
Diabetes	8,278 (14.2)	12,551 (22.1)	0<.001
Dyslipidemia	14,828 (25.5)	21,263 (37.5)	0<.001
Polycystic ovary syndrome	2,033 (3.5)	960 (1.7)	0<.001
**Duration of tamoxifen treatment**			
Never	−	56,679 (100)	
<1 year	23,470 (40.3)	−	
1–3 years	21,808 (37.5)	−	
3–5 years	8,954 (15.4)	−	
≥5 years	3,995 (6.8)	−	
**Gynecologic presentation**			
Abnormal bleeding	33,897 (58.2)	25,462 (44.9)	0<.001
Pain	30,033 (51.6)	26,422 (46.6)	0<.001
Diagnosis of endometrial lesions	5,388 (9.3)	4,627 (8.2)	0<.001
**Uterine cancer**	238 (0.4)	69 (0.1)	0<.001
Endometrioid adenocarcinoma	197 (82.8)	44 (63.8)	0.001
Non-endometrioid adenocarcinoma	29 (12.2)	16 (23.2)	
Uterine sarcoma and other carcinomas	12 (5.0)	9 (13.0)	
**Interval between initial breast cancer and endometrial cancer diagnosis** (years)	3.58 (2.06–4.91)	2.58 (1.61–4.48)	0.062
**Hysterectomy**	1,685 (2.9)	552 (1.0)	0<.001
**Death**	4,338 (7.5)	7,656 (13.5)	0<.001

BMI = body-mass index, Death = all causes of death.

Values are presented as median, range (25th-75th percentile), if no otherwise specified.

a Chi-square test or Fisher's exact tests were used for categorical variables and continuous variables were analyzed using Wilcoxon rank-sum test. Categorical variables with “Unknown” were excluded from chi-square analysis.

b Obesity classification according to Asian-Pacific cutoff points: underweight (<18.5 kg/m^2^), normal weight (18.5–22.9 kg/m^2^), overweight (23–24.9 kg/m^2^), and obese (≥25 kg/m^2^).

There were significant differences in the stages of breast cancer at diagnosis (*P* < 0.001). The proportion of breast cancer patients with CIS was higher in tamoxifen users than in non-users (18.8 % vs. 9.4 %, *P* < 0.001). The median age at breast cancer diagnosis was also lower in tamoxifen users (49 vs. 58 years, *P* < 0.001). The age distribution also displayed a greater proportion of women under 50 years of age (aged < 40 and 40–49) among tamoxifen users. The proportion of breast cancer patients by the calendar period of diagnosis (2011–2013, 2014–2016, and 2017–2019) decreased over time in tamoxifen users (35.6 %, 33.4 %, and 31.0 %), but it increased in non-users (21.9 %, 32.8 %, and 45.3 %) (*P* < 0.001). The treatment modalities for breast cancer, including surgery, radiotherapy, chemotherapy, hormonal therapy, oophorectomy, immunotherapy, targeted therapy, and palliative care, differed significantly between tamoxifen users and non-users. A higher proportion of breast cancer patients received surgery (92.2 % vs. 85.3 %, *P* < 0.001), radiation (56.8 % vs.48.5 %, *P* < -0.001), hormonal therapy (89.3 % vs. 45.0 %, *P* < 0.001) and oophorectomy (0.1 % vs.0.06 %, *P* = 0.007) among tamoxifen users (Table 1).

Among tamoxifen users, the majority (77.8 %) took tamoxifen for less than 3 years, and only 6.8 % had taken it for more than 5 years (Table 2). A medical history of hypertension (40.2 % vs. 26.1 %, *P* < 0.001), diabetes (22.1 % vs. 14.2 %, *P* < 0.001), or dyslipidemia (37.5 % vs. 25.5 %, *P* < 0.001) was higher in tamoxifen non-users than in tamoxifen users. A history of polycystic ovary syndrome (3.5 % vs. 1.7 %, *P* < 0.001), diagnosis of endometrial lesions (9.3 % vs. 8.2 %, *P* < 0.001), presentation of abnormal bleeding (58.2 % vs. 44.9 %, *P* < 0.001), and pain (51.6 % vs. 46.6 %, *P* < 0.001) were more common in tamoxifen users than in non-users. There were 238 (0.4 %) uterine cancer cases among the tamoxifen users and 69 (0.1 %) among non-users. The hysterectomy rate was also higher in tamoxifen users (2.9 % vs. 1.0 %, *P* < 0.001). The interval between the detection of breast and uterine cancers was not significantly different between tamoxifen users and non-users (median, 3.58 and 2.58 years, respectively, *P* = 0.06). Among uterine cancer cases, endometrioid adenocarcinoma was the most common subtype, followed by non-endometrioid adenocarcinoma, in both tamoxifen users and non-users.

**Fig. 2:**
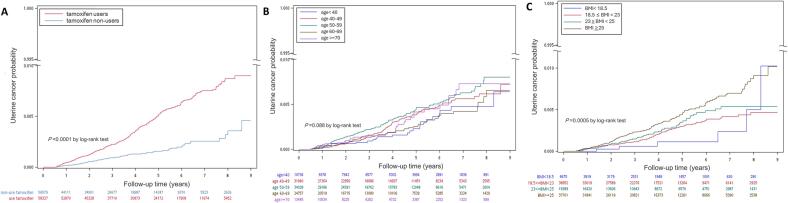
Probability of metachronous uterine cancer over follow-up period in patients with breast cancer by (A) tamoxifen use, (B) age at diagnosis and (C) body mass index (BMI).

Uterine cancer incidence was collected until 31 December 2019 and the median follow-up was 3.48 years (range 1.63 to 5.83). The total follow-up period was 255,034 person-years for tamoxifen users and 184,027 person-years for non-users. A total of 307 cases of uterine cancer were diagnosed during this period (238 in tamoxifen users and 69 in non-users) **(**Fig. 2A). The cumulative incidence of uterine cancer was significantly higher in tamoxifen users than in non-users (*P* < 0.001, log-rank test). Fig. 2B showed the cumulative incidence for each age category (aged < 40, 40–49, 50–59, 60–69, and 70 years or older). The incidence of uterine cancer was the highest in women aged 50–59 years and the cumulative incidence began to converge after 6 years (*P* = 0.088, log-rank test). Fig. 2C shows the number of uterine cancers diagnosed in women with breast cancer, based on different weight categories. BMI was associated with uterine cancer risk (*P* < 0.001, log-rank test), and a significant trend of increased risk was observed with higher BMI values.

Our analysis demonstrated that metachronous uterine cancer occurred especially in the initial 3 years of tamoxifen use. The incidence of newly diagnosed uterine cancer in patients receiving tamoxifen for less than 1 year was 105.52 cases per 100,000 person-years, that in patients receiving tamoxifen for 1–3 years was 111.62 cases per 100,000 person-years, that in patients receiving tamoxifen for 3–5 years was 58.19 cases per 100,000 person-years, and that in patients receiving tamoxifen for over 5 years was 62.12 cases per 100,000 person-years. The logistic regression model of the risk factors for uterine cancer in the cohort is shown in Table 3. Univariate analysis revealed that the significant risk factors for uterine cancer included age 50–59 years at diagnosis of breast cancer (HR 1.56, 95 % confidence interval (CI) 1.00–2.42, *P* = 0.04), stage at diagnosis of breast cancer, BMI (HR 1.06, 95 % CI 1.04–1.08, *P* < 0.001), a medical history of polycystic ovary syndrome (HR 2.14, 95 % CI 1.25–3.65, *P* = 0.005), and tamoxifen use (HR 2.35, 95 % CI 1.79–3.07, *P* < 0.001). Patients with invasive breast cancer at all stages (I–IV) had a lower risk of developing uterine cancer than those with CIS. The risk of uterine cancer showed an increasing trend as BMI increased from underweight to obesity. In multivariate logistic regression analysis, stage III at diagnosis of breast cancer (HR 0.45, 95 % CI 0.27–0.75, *P* = 0.002), BMI ≥ 25 (HR 2.46, 95 % CI 1.07–5.64, *P* = 0.03), and tamoxifen use were independent risk factors for uterine cancer. A significantly increased risk was observed in tamoxifen users. Substantial risk for uterine cancer was observed in patients with < 1 year and 1–3 years of use (HR 3.06, 95 % CI 2.14–4.39 for < 1 year; HR 3.03, 95 % CI 2.15–4.28 for 1–3 years), while longer use did not correlate with a proportionally higher risk (HR 1.61, 95 % CI 1.01–2.57, *P* = 0.04 for 3–5 year; HR 1.77, 95 % CI 1.00–3.13, *P* = 0.05 for 5 years or longer).

**Table 3:** Risk of uterine cancer in 114,906 patients with breast cancer from 2011 to 2019 in Taiwan.

Variable	*n*	Person-years	Uterine cancer	Uterine cancer Incidence a	Univariate			Multivariate		
					HR	95 % CI	*P*	HR	95 % CI	*P*
**Age at diagnosis of breast cancer**	114,906	438,941	307	69.94	1.00	0.99–1.01	0.68	1.01	1.00–1.02	0.11
<40	10,736	44,125	24	54.39	ref					
40–49	31,940	127,121	90	70.80	1.31	0.83–2.05	0.24			
50–59	34,028	134,411	113	84.07	1.56	1.00–2.42	0.04			
60–70	24,757	89,868	49	54.52	1.03	0.63–1.68	0.89			
≥70	13,445	43,562	31	71.16	1.38	0.81–2.36	0.23			
**Stage of breast cancer**										
0	16,333	64,842	67	103.33	ref			ref		
I	33,871	136,161	90	66.10	0.64	0.47–0.88	0.005	0.73	0.52–1.02	0.06
II	36,328	142,406	104	73.03	0.71	0.52–0.97	0.028	0.80	0.57–1.11	0.18
III	14,753	55,471	22	39.66	0.39	0.24–0.63	0<.001	0.45	0.27–0.75	0.002
IV	7,066	16,322	5	30.63	0.35	0.14–0.86	0.02	0.36	0.13–1.00	0.05
**BMI** (kg/m^2^)	100,771	385,953	261	67.62	1.06	1.04–1.08	0<.001			
<18.5	4,687	17,248	6	34.79	ref			ref		
18.5–22.9	38,971	151,207	80	52.91	1.50	0.66–3.44	0.33	1.48	0.65–3.40	0.35
23–24.9	19,402	74,892	48	64.09	1.82	0.78–4.25	0.16	1.80	0.77–4.23	0.17
≥25	37,711	142,548	127	89.09	2.55	1.12–5.77	0.02	2.46	1.07–5.64	0.03
**History of disease at breast cancer diagnosis**										
Hypertension	37,992	142,090	114	80.23	1.25	0.99–1.57	0.06	1.25	0.91–1.72	0.17
Diabetes	20,829	76,651	49	63.93	0.91	0.67–1.23	0.53	0.88	0.61–1.28	0.50
Dyslipidemia	36,091	135,702	90	66.32	0.94	0.73–1.20	0.60	0.73	0.53–1.01	0.05
Polycystic ovary syndrome	2,993	9,997	14	140.05	2.14	1.25–3.65	0.005	1.65	0.84–3.25	0.14
**Tamoxifen treatment**	58,227	255,034	238	93.32	2.35	1.79–3.07	0<.001			
Never	56,679	184,207	69	37.46	ref			ref		
<1 year	23,470	74,869	79	105.52	2.83	2.05–3.91	0<.001	3.06	2.14–4.39	0<.001
1–3 years	21,808	99,444	111	111.62	2.82	2.09–3.81	0<.001	3.03	2.15–4.28	0<.001
3–5 years	8,954	53,276	31	58.19	1.37	0.90–2.10	0.14	1.61	1.01–2.57	0.04
≥5 years	3,995	27,366	17	62.12	1.41	0.83–2.40	0.20	1.77	1.00–3.13	0.05

HR: hazard ratio, CI: confidence interval.

a Uterine cancer incidence rate per 100,000 person-years.

In our cohort, almost all patients with breast cancer receiving tamoxifen underwent regular gynecological ultrasound screening to monitor endometrial changes. The diagnosis of endometrial lesions is coded based on imaging findings of sonography or office hysteroscopy in the majority of cases, and invasive diagnostic biopsy is generally arranged for histopathological approval, even in women without any clinical symptoms (e.g., bleeding or pain). Therefore, a subgroup analysis stratified by abnormal uterine bleeding and the diagnosis of endometrial lesions was performed (Supplementary Table 2). In women presenting with abnormal uterine bleeding and diagnosis of endometrial lesions, BMI (HR 2.97, 95 % CI 1.30–6.82, *P* = 0.01) and tamoxifen use (HR 1.08, 95 % CI 1.01–1.16, *P* = 0.02) were significant risk factors for uterine cancer. In patients presenting with abnormal uterine bleeding but without diagnosis of endometrial lesions, the stage at diagnosis of breast cancer, BMI (HR 2.95, 95 % CI 1.70–5.09, *P* < 0.001), and tamoxifen use (HR 1.10, 95 % CI 1.06–1.14, *P* < 0.001) were significant risk factors for uterine cancer. In patients diagnosed with endometrial lesions but without abnormal uterine bleeding, tamoxifen did not affect the risk of uterine cancer. In women without both diagnosis of endometrial lesions and abnormal uterine bleeding, BMI (HR 2.78, 95 % CI 1.78–4.33, *P* < 0.001) and tamoxifen use (HR 1.05, 95 % CI 1.01–1.08, *P* = 0.001) were two significant risk factors for uterine cancer.

## 4 Discussion

Our study showed that a higher risk of metachronous uterine cancer occurred in patients with breast cancer receiving tamoxifen, especially in the initial 3 years. Higher BMI and tamoxifen use were independent risk factors for metachronous uterine cancer in breast cancer patients who presented with abnormal uterine bleeding. Risk models from population-based cohorts for the prediction of uterine cancer have been developed, and the risk factors in these models include BMI, parity, age at menarche and at menopause, smoking, and oral contraceptive use. (Pfeiffer et al., 2013, Hüsing et al., 2016, Kitson et al., 2023) However, a predictive risk model for uterine cancer in patients with breast cancer is not currently available. Tamoxifen, a selective estrogen receptor modulator, is extensively used as a hormonal treatment for metastatic breast cancer and as an adjuvant treatment for high-risk breast cancer. Common side effects of tamoxifen include sexual dysfunction and vasomotor disturbances, but the most concerning gynecologic effects are endometrial polyp, endometrial hyperplasia, and uterine cancer. (Mourits et al., 2001) Relative risk estimation of uterine cancer ranged from 1.3 to 7.5 in previous studies. (Sung et al., 2021, Molina-Montes et al., 2015, Molina-Montes et al., 2013) The incidence of uterine cancer in this study was similar to that of tamoxifen treated patients with breast cancer observed in previous cohort studies. (Chu et al., 2019, Chen et al., 2014) However, different results were observed across studies investigating the duration of tamoxifen use and risk of uterine cancer. (Davies et al., 2013, Ghanavati, 2023) The duration of tamoxifen use was not positively correlated with the risk of uterine cancer in our analysis, which might be due to the switch from tamoxifen to other adjunctive hormone therapies after the occurrence of uterine cancer. Some studies have suggested that prolonged tamoxifen exposure may alter estrogen receptor expression, reducing its proliferative effect on the endometrium with a lower relative risk over time. (Deligdisch, 2000) Additionally, tamoxifen may promote the growth of pre-existing occult lesions, contributing to the observed differences in risk over time.

Our study indicated that a high BMI and tamoxifen use were significant risk factors for metachronous uterine cancer in patients presenting with abnormal uterine bleeding, irrespective of the clinical diagnosis of endometrial lesions. Patients with breast cancer receiving tamoxifen are generally informed about the risks, but there are no clear recommendations regarding screening for uterine diseases in these patients. Screening options such as sonography, D&C, endometrial biopsy, and serum biomarker tests have been investigated, but there are controversies in the cost-effectiveness of these methods. (Sroczynski et al., 2020) Berliere *et al*. identified a high-risk group by pretreatment uterine evaluation, and endometrial resection did not protect against the subsequent development of endometrial cancer in patients on tamoxifen. (Berliere et al., 2000) Routine screening increased the rate of incidental findings, resulting in unnecessary surgical procedures and complications. A recent study by Choi *et al* reported higher frequencies of invasive endometrial procedures in tamoxifen users than in non-users, and invasive endometrial evaluation was conducted more than 46 times to find one uterine cancer in patients aged < 40 years. (Choi et al., 2021) Ultrasonographic endometrial assessments, including thickness, echotexture, border, and presence of fluid, have been widely used in tamoxifen users, although most of them do not experience clinical signs suggestive of hyperplasia or malignancy. However, ultrasonographic findings in tamoxifen users might be misinterpreted because of a false expansion of endometrium and distortion of myometrial border by cystic spaces in the subsendometrial layer. (Bornstein et al., 1994) The specificity of endometrial measurement can be further improved by saline infusion hysterosonography. (Develioğlu et al., 2004) Endometrial thickness, presence of mass, and vaginal bleeding were reported to be predictors of abnormal histological findings among postmenopausal tamoxifen users. (Franchi et al., 1999) A cutoff ranging from 5 to 10 mm for endometrial thickness has been advocated by some investigators as diagnostic of endometrial pathology. (Develioğlu et al., 2004, Franchi et al., 1999, Strauss et al., 2000) A dilemma exists as to the optimal cutoff value of thickness in both symptomatic and asymptomatic women on tamoxifen, which requires intervention. There is no clear consensus on whether the time to endometrial cancer diagnosis is shorter among tamoxifen users. The increased risk of endometrial cancer in tamoxifen users often leads to closer gynecological surveillance, which can lead to earlier detection in some cases. However, factors such as misattribution of symptoms, endometrial thickening, and histologic subtype differences may delay diagnosis in others.

The strengths of this study include the investigation of risk factor prediction using a nationwide population database. To aid clinical interpretation, we defined categorical BMI risk groups according to the Asia-Pacific classification and measures of tamoxifen utilization based on the duration of treatment. Another advantage is that our study incorporated common endometrial pathologies associated with tamoxifen exposure and clinical symptoms, such as abnormal bleeding, for analysis. The limitations were the lack of data, such as menopausal status and cumulative dose of tamoxifen. The cumulative dose of tamoxifen has been correlated with the risk of uterine cancer in previous studies; however, the duration of tamoxifen administration may not accurately reflect the actual dose.

In conclusion, breast cancer patients taking tamoxifen had a significantly higher risk of metachronous uterine cancer than those not taking tamoxifen, especially in the initial 3 years. High BMI and abnormal uterine bleeding significantly impacted the risk of metachronous uterine cancer, irrespective of the clinical diagnosis of endometrial lesion. This indicates that routine ultrasound is not currently recommended for the detection of uterine cancer. Existing evidence does not support its efficacy as a screening tool and routine use may lead to unnecessary interventions. Gynecologic assessment for metachronous uterine cancer should focus on breast cancer patients with high BMI, tamoxifen use, and abnormal uterine bleeding to avoid unnecessary invasive procedures. Notably, in the clinical care of breast cancer patients, the analysis in this study showed that a substantial proportion of breast cancer patients who did not use tamoxifen also had common risk factors for endometrial cancer, such as obesity (35.1 %) and abnormal bleeding (44.9 %). These patients should also be referred to gynecologists for endometrial evaluation.

## CRediT authorship contribution statement

**Yi-Jou Tai:** Writing – original draft, Validation, Investigation, Formal analysis, Conceptualization. **Chung-Chen Lee:** Software, Resources, Formal analysis, Data curation. **Yong-Chen Chen:** Validation, Software, Resources, Formal analysis. **San-Lin You:** Supervision, Methodology, Data curation, Conceptualization. **Ying-Cheng Chiang:** Writing – review & editing, Supervision, Investigation, Conceptualization.

## Funding

This research received no funding.

## Declaration of competing interest

The authors declare that they have no known competing financial interests or personal relationships that could have appeared to influence the work reported in this paper.

## Data Availability

Data will be made available on request.
