# Supplementary material for: Warning factors of metachronous uterine cancer in patients with breast cancer: a real-world nationwide cohort study
Source: Gynecol Oncol Rep. 2025 Apr 5;59:101732. doi: 10.1016/j.gore.2025.101732 (PMC12013394; doi:10.1016/j.gore.2025.101732)
Supplement: Supplementary Data 1 [file mmc1.docx]

**Supplementary Table 1**

|  | **Definitions** | | **Code** |
| --- | --- | --- | --- |
| **Patients** | | | |
| Breast cancer | Defined from diagnosis in NHI Taiwan Cancer Registry ^a^ | ICD-9-CM: 174  ICD-10-CM : C50 | |
| **Exclusion** | | | |
| Other cancer | Defined from diagnosis in NHI Taiwan Cancer Registry ^a^ | ICD-9-CM : 140-208  (exclude 174)  ICD-10-CM : C00-C97 (exclude C50) | |
| Hysterectomy | Defined from diagnosis with surgery ^b^ | NHI Surgery Code:  80403B、80404C、80411B、80412B、80413B、80414B、80416B、80421B、80429B、80430B、81005C、81029C、97022B、97027C、97037B、N26034、80026B、80027B、78020B、80418B、80417B、80424B、N26037 | |
| **Comorbidities** | | | |
| Tamoxifen | Defined from diagnosis with prescription of statins drugs ^d^ | | ATC code : L02BA01 |
| Hypertension | Defined from diagnosis ^c^ | | ICD-9-CM : 401-405  ICD-10-CM : I10-I13、I15 |
| Diabetes | Defined from diagnosis ^c^ | | ICD-9-CM : 250  ICD-10-CM : E08-E11、E13 |
| Dyslipidemia | Defined from diagnosis ^c^ | | ICD-9-CM : 272.0-272.4  ICD-10-CM : E78.0-E78.5 |
| Polycystic ovary syndrome | Defined from diagnosis ^d^ | | ICD-9-CM : 256.4  ICD-10-CM : E28.2 |
| Abnormal bleeding | Defined from diagnosis ^c^ | | ICD-9-CM : 626.8、626.9、627.1、621.4、626.4  ICD-10-CM : N93.8、N93.9、N95.0、N85.7、N92.5、N92.6 |
| Endometrial lesion | Defined from diagnosis ^c^ | | ICD-9-CM : 621.0、621.3、621.8、219.1、239.5  ICD-10-CM : N84.0、N85.00、N85.01、N85.8、D26.1、D49.5 |
| **Clinical outcomes** | | | |
| Uterine cancer | Defined from diagnosis in NHI Taiwan Cancer Registry ^e^ | | ICD-9-CM : 182  ICD-10-CM : C54 |
| Subtype of uterine cancer | | | |
| Endometrioid adenocarcinoma | Defined from diagnosis in NHI Taiwan Cancer Registry ^f^ | | ICD-O-3 M-code : 8140、8260、8262、8263、8323、8380、8382、8383、8384、8570 |
| Non-endometrioid adenocarcinoma | Defined from diagnosis in NHI Taiwan Cancer Registry ^f^ | | ICD-O-3 M-code : 8310、8441、8460、8461、8480、8481、8560、8950、8951、8980 |
| Uterine sarcoma | Defined from diagnosis in NHI Taiwan Cancer Registry ^f^ | | ICD-O-3 M-code : 8714、8800、8802、8805、8851、8890、8891、8896、8920、8930、8931、8933、8935、9040 |
| Other carcinomas | Defined from diagnosis in NHI Taiwan Cancer Registry ^f^ | | ICD-O-3 M-code : 8000、8010、8013、8020、8030、8033、8041、8050、8070、8071、8072、8246、8255、8381、8574、8801、8900、8901、8963、9100、9104、9105 |

NHI: National Health Insurance, ATC: Anatomical Therapeutic Chemical, M-code: Morphology code

^a^ Cancer was established based on at least one time NHI Taiwan cancer registry record of ICD-9 codes in the database.

^b^ Surgery was established based on at least one time inpatient records of NHI surgery code in the database.

^c^ To ensure accuracy, comorbidities were established based on at least three times inpatient and outpatient records of ICD-9 codes in the database.

^d^ Comorbidities and drugs were established based on at least one time inpatient and outpatient records of ICD-9 codes in the database.

^e^ Uterine cancer was established based on at least one time NHI Taiwan cancer registry records of ICD-9 and ICD-10 codes in the database.

^f^ Histologic subtype of uterine cancer was defined based on NHI Taiwan cancer registry records of ICD-O-3 Morphology code in the database.
