# Supplementary material for: Warning factors of metachronous uterine cancer in patients with breast cancer: a real-world nationwide cohort study
Source: Gynecol Oncol Rep. 2025 Apr 5;59:101732. doi: 10.1016/j.gore.2025.101732 (PMC12013394; doi:10.1016/j.gore.2025.101732)
Supplement: Supplementary Data 2 [file mmc2.docx]

**Supplementary Table 2.** Risk of uterine cancer stratified by gynecologic presentations in 114,906 patients with breast cancer from 2011 to 2019 in Taiwan

| *With abnormal bleeding* | With endometrial lesion(s) | | | | | | Without endometrial lesion(s) | | | | | |
| --- | --- | --- | --- | --- | --- | --- | --- | --- | --- | --- | --- | --- |
|  | Univariate | | | Multivariate | | | Univariate | | | Multivariate | | |
|  | HR | 95% CI | *P* | HR | 95% CI | *P* | HR | 95% CI | *P* | HR | 95% CI | *P* |
| **Age at diagnosis of BC** | 1.02 | 0.99-1.05 | .20 | 1.03 | 0.99-1.07 | .19 | 1.00 | 0.99-1.02 | .66 | 1.02 | 1.00-1.05 | 0.12 |
| **Stage at diagnosis of BC** |  |  |  |  |  |  |  |  |  |  |  |  |
| Early (stage 0-II) | ref |  |  | ref |  |  | ref |  |  | ref |  |  |
| Advanced (stage III, IV) | 0.33 | 0.08-1.36 | .12 | 0.42 | 0.10-1.75 | .23 | 0.56 | 0.29-1.07 | .07 | 0.43 | 0.20-0.94 | .033 |
| **BMI** (kg/m^2^) | 1.09 | 1.03-1.16 | .004 | 2.97 | 1.30-6.82 | .01 | 1.09 | 1.05-1.13 | <.001 | 2.95 | 1.70-5.09 | <.001 |
| **History of disease at BC diagnosis** | | | | | | |  |  |  |  |  |  |
| Hypertension | 1.86 | 1.05-3.30 | .03 | 1.05 | 0.48-2.33 | .89 | 1.00 | 0.66-1.54 | .98 | 0.85 | 0.48-1.49 | .57 |
| Diabetes | 1.56 | 0.78-3.13 | .21 | 1.57 | 0.67-3.67 | .30 | 1.22 | 0.73-2.03 | .44 | 1.14 | 0.58-2.25 | .69 |
| Dyslipidemia | 1.27 | 0.70-2.29 | .43 | 0.82 | 0.38-1.79 | .62 | 0.86 | 0.56-1.32 | .48 | 0.59 | 0.33-1.07 | .08 |
| Polycystic ovary syndrome | 2.09 | 0.83-5.29 | .11 | 1.09 | 0.26-4.68 | .90 | 1.73 | 0.85-3.55 | .13 | 1.49 | 0.64-3.48 | .35 |
| **Tamoxifen use** | 2.46 | 1.19-5.08 | .01 | 1.08 | 1.01-1.16 | .02 | 2.40 | 1.52-3.77 | <.001 | 1.10 | 1.06-1.14 | <.001 |

| *No abnormal bleeding* | With endometrial lesion(s) | | | | | | Without endometrial lesion(s) | | | | | |
| --- | --- | --- | --- | --- | --- | --- | --- | --- | --- | --- | --- | --- |
|  | Univariate | | | Multivariate | | | Univariate | | | Multivariate | | |
|  | HR | 95% CI | *P* | HR | 95% CI | *P* | HR | 95% CI | *P* | HR | 95% CI | *P* |
| **Age at diagnosis of BC** | 1.10 | 1.03-1.18 | .007 | 1.09 | 1.00-1.19 | .05 | 1.00 | 0.99-1.02 | .87 | 1.01 | 0.99-1.03 | .16 |
| **Stage at diagnosis of BC** |  |  |  |  |  |  |  |  |  |  |  |  |
| Early (stage 0-II) | ref |  |  | ref |  |  | ref |  |  | ref |  |  |
| Advanced (stage III, IV) | 1.56 | 0.17-13.97 | .69 | 1.74 | 0.18-16.57 | .62 | 0.54 | 0.31-0.95 | .03 | 0.67 | 0.38-1.18 | .16 |
| **BMI** (kg/m^2^) | 1.01 | 0.83-1.22 | .93 | 0.38 | 0.04-3.63 | .39 | 1.05 | 1.01-1.08 | .006 | 2.78 | 1.78-4.33 | <.001 |
| **History of disease at BC diagnosis** | | | | | | |  |  |  |  |  |  |
| Hypertension | 6.38 | 0.71-57.04 | .09 | 3.28 | 0.28-39.08 | .34 | 1.31 | 0.93-1.85 | .12 | 1.44 | 0.90-2.30 | .13 |
| Diabetes | 0.95 | 0.11-8.54 | .96 | 0.39 | 0.04-4.01 | .43 | 0.66 | 0.41-1.05 | .07 | 0.64 | 0.37-1.09 | .09 |
| Dyslipidemia | 2.36 | 0.39-14.14 | .34 | 1.09 | 0.15-7.75 | .92 | 0.88 | 0.61-1.26 | .48 | 0.75 | 0.47-1.18 | .20 |
| Polycystic ovary syndrome |  |  |  |  |  |  | 2.16 | 0.30-15.45 | .44 | 2.21 | 0.31-16.05 | .43 |
| **Tamoxifen use** | 0.20 | 0.02-1.82 | .15 | 0.96 | 0.76-1.21 | .71 | 2.47 | 1.66-3.68 | <.0001 | 1.05 | 1.01-1.08 | .01 |

HR: hazard ratio, CI: confidence interval. BC: breast cancer
